# A Novel Approach to Delayed-Start Analyses for Demonstrating Disease-Modifying Effects in Alzheimer’s Disease

**DOI:** 10.1371/journal.pone.0119632

**Published:** 2015-03-17

**Authors:** Hong Liu-Seifert, Scott W. Andersen, Ilya Lipkovich, Karen C. Holdridge, Eric Siemers

**Affiliations:** Eli Lilly and Company, Indianapolis, Indiana, United States of America; Georgetown University Medical Center, UNITED STATES

## Abstract

One method for demonstrating disease modification is a delayed-start design, consisting of a placebo-controlled period followed by a delayed-start period wherein all patients receive active treatment. To address methodological issues in previous delayed-start approaches, we propose a new method that is robust across conditions of drug effect, discontinuation rates, and missing data mechanisms. We propose a modeling approach and test procedure to test the hypothesis of noninferiority, comparing the treatment difference at the end of the delayed-start period with that at the end of the placebo-controlled period. We conducted simulations to identify the optimal noninferiority testing procedure to ensure the method was robust across scenarios and assumptions, and to evaluate the appropriate modeling approach for analyzing the delayed-start period. We then applied this methodology to Phase 3 solanezumab clinical trial data for mild Alzheimer’s disease patients. Simulation results showed a testing procedure using a proportional noninferiority margin was robust for detecting disease-modifying effects; conditions of high and moderate discontinuations; and with various missing data mechanisms. Using all data from all randomized patients in a single model over both the placebo-controlled and delayed-start study periods demonstrated good statistical performance. In analysis of solanezumab data using this methodology, the noninferiority criterion was met, indicating the treatment difference at the end of the placebo-controlled studies was preserved at the end of the delayed-start period within a pre-defined margin. The proposed noninferiority method for delayed-start analysis controls Type I error rate well and addresses many challenges posed by previous approaches. Delayed-start studies employing the proposed analysis approach could be used to provide evidence of a disease-modifying effect. This method has been communicated with FDA and has been successfully applied to actual clinical trial data accrued from the Phase 3 clinical trials of solanezumab.

## Introduction

### Background on Alzheimer’s disease

Alzheimer’s disease (AD) is an age-related neurodegenerative disorder characterized by a progressive decline in cognitive function. The course of AD involves progressive memory loss, behavioral decline, gait and motor disturbances, and the inability to perform activities of daily living, eventually leading to complete dependence on a caregiver, usually followed by nursing home care [1,2]. AD is a major and rapidly increasing public health concern: over 30 million individuals worldwide suffer from AD and this number is projected to quadruple by 2050.[3] AD has been reported to be the 3rd leading cause of death in US [4]. The cost of treating AD is an increasing burden to society; in 2010, the global cost of treating dementia, including dementia due to AD and other causes, was greater than US$600 billion [5]. Currently approved treatments attenuate the symptoms of AD, but have not been shown to affect the underlying pathology [6]. With this impending global public health crisis, treatments that prevent onset or slow progression of AD are clearly needed.

### Background on delayed-start design

Delayed-start designs were proposed by Leber [7] as a possible strategy to demonstrate a disease-modification drug effect, which is considered an effect that slows the progression of disease by modifying the underlying biological pathology, rather than only attenuating symptoms. Development of these designs was motivated by a study of tacrine in patients with probable AD [8] in which, after a 6-week, double-blind, placebo-controlled phase, patients originally randomized to placebo were switched to tacrine under open-label conditions. Six weeks later, the ADAS-Cog scores of the patients switched from placebo to tacrine were virtually the same as those randomized to tacrine at the beginning of the double-blind phase—that is, after 6 weeks, patients who started tacrine late “caught up to” those who had been on tacrine continuously for 12 weeks.

**Fig 1:**
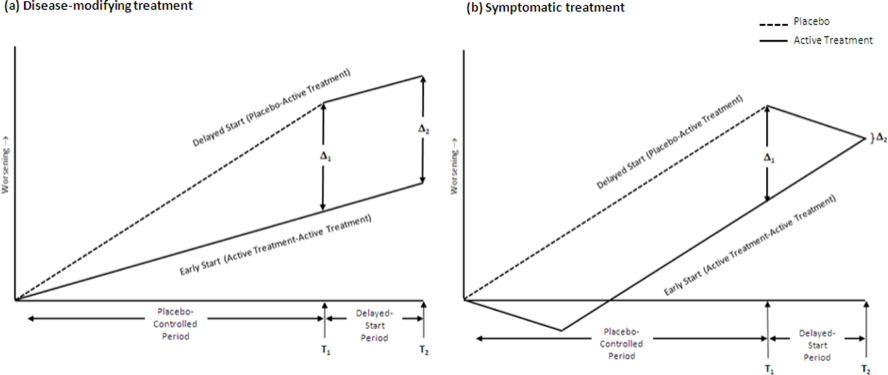
Delayed-start design under conditions of (a) a disease-modifying treatment effect and (b) a symptomatic treatment effect. Δ_1_ represents the initial treatment effect at the end of the placebo-controlled period (T_1_) and Δ_2_ represents the treatment effect at the end of the delayed-start period (T_2_). The hypothetical results for a symptomatic treatment (b) assume that the time to peak treatment effect is equal to the duration of the delayed-start period.

Leber [7] defined a delayed-start, or randomized-start, study as one in which patients are randomized to the same active treatment but at different times, resulting in two treatment periods: a placebo-controlled period followed by a delayed-start period (Fig. 1). During the placebo-controlled period, patients are randomized to either an active treatment or placebo. During the delayed-start period, placebo patients are switched to receive the active treatment and thus become delayed-start patients. Active-treatment patients continue to receive active treatment during the delayed-start period and are labeled as early-start patients. Thus, in a delayed-start study, patients are randomized at the beginning of the placebo-controlled period to be either early- or delayed-start patients. During the length of the study (that is, both the placebo-controlled and delayed-start periods), all patients and study personnel are blinded to each patient’s randomization to the early-start or late-start treatment group.

The rationale of a delayed-start design is that under the null hypothesis, when the active drug has a purely symptomatic effect and has no effect on neuropathologic process, a delay in administration should have no lasting effect on patients. Thus the delayed-start patients are expected to catch up to early-start patients (Fig. 1b). However, if the effect were not purely symptomatic and had altered the underlying progression, the delayed-start patients could not possibly overcome the losses sustained during the delay period (Fig. 1a). In other words, if the delayed-start patients do not “catch up” with the early-start patients (that is, the outcome for the delayed-start patients does not become similar in magnitude to that of the early-start patients), a conclusion can be drawn that the treatment is not purely symptomatic and has modified the underlying course of the disease, representing a disease-modifying effect. Such finding includes the possibility that the drug may have both symptomatic and disease-modifying effects. Demonstrating a disease-modifying effect would imply a sustained benefit of starting such drugs early.

### Current analytical approaches for delayed-start design

The application of a delayed-start design has been limited due to operational challenges, as well as the absence of a robust statistical method for the analysis and interpretation of data. Two similar approaches for sequential testing have been proposed for the analysis of delayed-start studies to ascertain potential disease-modifying effects. Bhattaram and colleagues [9] proposed the following tests comparing early- and delayed-start treatment groups for the analysis of Parkinson’s disease (PD) studies: (1) the difference in slopes in the placebo-controlled period, (2) the difference in mean change from baseline to the end of the delayed-start period on the primary outcome measure, and (3) noninferiority comparison of slopes in the delayed-start period. This method recommends the exclusion of data from the first 12 weeks of each study period to enable the testing of the drug effect on slope. Zhang and colleagues [10] proposed the following tests comparing early- and delayed-start treatment groups for the analysis of AD studies: (1) the difference in mean change from baseline on the primary outcome measure at the end of the placebo-controlled period, (2) the difference in mean change from baseline on the primary outcome measure at the end of the delayed-start period, and (3) noninferiority comparison of slopes at the end of the delayed-start period.

The approach proposed by Bhattaram et al. [9] has been implemented in the Attenuation of Disease Progression with Azilect Given Once-daily (ADAGIO) study to support a disease-modification claim for rasagiline, a monoamine oxidase type B inhibitor approved for the symptomatic treatment of PD [11]. A US Food and Drug Administration (FDA) advisory committee meeting was held and the committee voted unanimously against a recommendation for the disease-modification claim because of insufficient evidence and several methodological issues around the analyses of the delayed-start period.

The ADAGIO trial used a noninferiority test of the slopes with a preselected noninferiority margin of 0.15 points. Among patients who received rasagiline 2 mg per day, there was no significant difference in the change in the primary outcome measure from baseline to the end of the delayed-start period between the early-start group and the delayed-start group; the delayed-start patients not only caught up with the early-start patients, but surpassed them. Conversely, noninferiority *was* demonstrated in the comparison of the rate of change from week 48 to week 72 between the early-start group and the delayed-start group [11]. This clearly contradictory outcome and conclusion stemmed from the noninferiority test procedure, which used an absolute noninferiority margin of 0.15 to compare the two slopes and did not effectively detect the actual differences between the two treatment groups, as both slopes had small magnitude.

Another critical issue raised at the ADAGIO FDA advisory committee was regarding excluding randomized patients and its implications. The study allowed patients to transition early to the delayed-start period before completing the placebo-controlled period. In testing their hypotheses 2 and 3, patients who had less than 24 weeks of treatment in the placebo-controlled period were excluded from the analyses. This resulted in excluding 15% of the randomized patients, leading to an imbalance in demographics and illness characteristics between the early-start and delayed-start patients.

Zhang’s method for noninferiority testing calls for testing the slopes immediately before the end of the delayed-start period. They suggested monthly cognitive assessments for the last 3 months before the end of the delayed-start period to evaluate the slopes in this 3-month window. While this suggestion is interesting, it may not be feasible to obtain monthly assessments using the ADAS-Cog (for example, because of the number of versions of the scale available, potential training effects, and increased patient burden). Further, conducting the noninferiority test based on the last 3 months of the delayed-start period would include only an unrandomized subset of the overall patient population still participating the last 3 months of the study [10]. This would lead to imbalance between the early-start and delayed-start patients who are available in the last 3 months of the delayed-start period and thus challenges in interpreting the results. Application of this method to actual clinical trial data or simulation work may help the field to better understand the performance of the proposal; however, to the best of our knowledge, such application has not been reported.

### Proposed improved analytical method for delayed-start design

In principle, we agree with the first two tests suggested by Zhang and colleagues (change from baseline at the end of the placebo-controlled period and change from baseline at the end of the delayed-start period) [10]. While both Bhattaram [9] and Zhang postulated similar noninferiority tests for the delayed-start period (the third test), we believe these tests, as suggested, have inherent drawbacks, and we propose an alternative methodology for the noninferiority test to overcome the issues.

We propose to conduct the noninferiority test by comparing the treatment difference at the beginning of the delayed-start period (Δ_1_) with the treatment difference at the end of the delayed-start period (Δ_2_) using a proportional margin [12, 13]. If the treatment difference at the end of the delayed-start period is greater than a prespecified proportion of the treatment difference at the beginning of the delayed-start period, then noninferiority is demonstrated.

Furthermore, discontinuations in both the placebo-controlled and delayed-start periods will lead to missing data and require appropriate statistical methodology. In particular, discontinuations in the placebo-controlled period driven by lack of efficacy or other treatment outcomes could cause selection bias for subsequent comparisons between the delayed-start and early-start patient groups in the delayed-start period. To address this potential issue, we propose to estimate the treatment outcomes and effects for the delayed-start period using a single likelihood-based mixed effects model for repeated measures (MMRM) including all randomized patients and all data from both placebo-controlled and delayed-start periods. This approach is consistent with the principled likelihood-based approach for analysis of repeated measures [14–20], yielding an estimate of the treatment outcomes as if all patients had stayed in the trial. MMRM has been shown to be a well-suited statistical method for estimating treatment effect in a longitudinal clinical trial when data are missing at random [21]. Generally, in well-designed clinical trials it is reasonable to assume that dropout patterns follow the missing at random mechanism, although data missing not at random cannot be ruled out [22].

Given the complexity of analysis of longitudinal data and lack of closed-form analytical solutions for the statistical implications of using different analysis models in combination with different noninferiority testing strategies, a simulation study was conducted to evaluate various potential test procedures to carry out the noninferiority tests. The most optimal procedure was then recommended based on systematic assessment of the statistical performance of the proposed methodologies. Additionally, these simulations compared the approach of modeling over all randomized patients versus modeling over only data from the delayed-start period. All simulations were conducted to ensure the proposed approach was robust across various conditions of underlying drug effect, discontinuation rates, and missingness mechanisms.

The proposed approach for delayed-start analysis was applied to the Phase 3 clinical trials of solanezumab for the treatment of AD; the results are reported here to demonstrate the application of the proposed methodologies.

## Methods

As noted above, several authors have proposed noninferiority testing procedures to analyze delayed-start data to demonstrate a disease-modifying effect. To mitigate the drawbacks to these specific tests as described above, we propose alternative modeling and noninferiority testing between the treatment difference at the end of the delayed-start period and the treatment difference at the end of the placebo-controlled period.

We propose the following noninferiority test where the noninferiority margin is equal to 50% of Δ_1_ [11].

$$H_{0}:\Delta_{2}-\Delta_{1}\leq -0.5\Delta_{1}\;\text{versus}\;H_{a}:\Delta_{2}-\Delta_{1}\leq -0.5\Delta_{1}$$

H_a_ is equivalent to Δ_2_ > (1–0.5)Δ_1_ = 0.5Δ_1_, which implies that the treatment effect at the end of the delayed-start period (Δ_2_) preserved more than 50% of the treatment effect after the placebo-controlled period (Δ_1_) or Δ_2_ did not lose more than 50% of Δ_1_.

### Candidate noninferiority approaches

To demonstrate that outcome scores from delayed-start patients do not approximate those from early-start patients at the end of the delayed-start period, we propose to conduct a noninferiority test to show that the treatment difference at the end of the delayed-start period (Δ_2_) preserved more than 50% of the treatment effect at the end of the placebo-controlled period (Δ_1_). The alternative hypothesis of this noninferiority test can be described as follows

$$\Delta_{2}-\Delta_{1}>-0.5\Delta_{1}\;\text{or}$$

$$\Delta_{2}>0.5\Delta_{1}\;\text{or}$$

$$\Delta_{2}-0.5\Delta_{1}>0$$

The noninferiority margin represents the proportion of initial treatment difference lost in the delayed-start period. Because the original alternative hypothesis (Δ_2_ –Δ_1_ > –0.5 Δ_1_) has estimates on both sides of the equation and there is no obvious solution to the test statistics, we selected several test procedures (Table 1) for consideration to perform the noninferiority test. Specifically, we explored several test procedures based on the lower bound of the 1-sided 90% confidence interval to identify the best approach to carry out the noninferiority test (Table 1). Clinical trial simulations using various scenarios and assumptions were run to compare the Type I error rates and power of the different test procedures.

**Table 1:** Candidate test procedures for noninferiority test.

	Lower Bound of 1-Sided 90% CI	Threshold
(1)	Δ_2_ –Δ_1_	> -0.5x the upper bound of 1-sided 90% CI for Δ_1_
(2)	Δ_2_ –Δ_1_	> -0.5x the estimate for Δ_1_
(3)	Δ_2_	> 0.5x the estimate for Δ_1_
(4)	Δ_2_–0.5 Δ_1_	> 0
(5)	Δ_2_ –Δ_1_	> -0.5x the lower bound of 1-sided 90% CI for Δ_1_

Note: For each procedure, the lower bound of the 1-sided 90% confidence interval must be greater than the specified threshold (right column).

### Clinical trial simulations

The clinical trial used in the simulations included two treatments, active and placebo, with 500 patients randomly assigned to each treatment. The simulated trials include an 18-month placebo-controlled phase and a 6-month delayed-start phase, with all postbaseline visits at 3-month intervals. Two scenarios of underlying true treatment effect were assumed with hypothetical treatment outcomes (Table 2). A disease-modifying treatment effect scenario (scenario 1) was constructed such that the two slopes in the delayed-start period were parallel to each other. That is, the treatment difference seen at the end of the placebo-controlled period (month 18) was equal to the treatment difference at the end of the delayed-start period (month 24). A symptomatic treatment effect scenario (scenario 2) was constructed such that the treatment effect seen at the end of the delayed-start period (month 24) was half of the treatment effect seen at the end of the placebo-controlled period. Two thousand trials were simulated for each of these two scenarios using the underlying mean structures shown in Table 2. The primary outcome measure used for the simulations was the Alzheimer’s Disease Assessment Scale-Cognitive subscale (ADAS-Cog_11_).

**Table 2:** Underlying mean ADAS-Cog scores for simulations in the disease-modifying treatment effect scenario (Scenario 1) and the symptomatic treatment effect scenario (Scenario 2).

	Months from Baseline								
	Baseline	3	6	9	12	15	18	21^a^	24^a^
Disease-Modifying Treatment Effect Scenario (Scenario 1)									
Active (raw)	22	22.9	23.8	24.7	25.6	26.5	27.4	28.3	29.2
Active (change)	0	0.9	1.8	2.7	3.6	4.5	5.4	6.3	7.2
Placebo (raw)	22	23.2	24.4	25.6	26.8	28	29.2	30.1	31
Placebo (change)	0	1.2	2.4	3.6	4.8	6	7.2	8.1	9
Standard deviation (raw)	9	10	11	12	13	14	15	16	17
Symptomatic Treatment Effect Scenario (Scenario 2)									
Active (raw)	22	22.9	23.8	24.7	25.6	26.5	27.4	28.3	29.2
Active (change)	0	0.9	1.8	2.7	3.6	4.5	5.4	6.3	7.2
Placebo (raw)	22	23.2	24.4	25.6	26.8	28	29.2	29.65	30.1
Placebo (change)	0	1.2	2.4	3.6	4.8	6	7.2	7.65	8.1
Standard deviation (raw)	9	10	11	12	13	14	15	16	17

^a^Assessments at months 21 and 24 occur during the delayed-start period.

The underlying error distribution in the linear repeated-measures model was multivariate normal. The assumed within-patient correlation and variance matrix was based on clinical trial data from an AD study and reflected increasing variance over time and diminishing correlations among visits as time between visits increased (data not shown).

For each of the two underlying treatment effect scenarios, SAS v9 was used to generate complete simulated trial data from a multivariate normal distribution with the means structure and correlation matrix detailed above (Table 2). Patient dropout was imposed on the complete simulated datasets according to three different mechanisms—missing completely at random (MCAR), missing at random (MAR), and missing not at random (MNAR)—at two different levels of patient dropout (25% and 40%) to create the final simulated clinical trial datasets to be used for analysis.

Missing completely at random was simulated by using a Bernoulli distribution with the probability of a patient dropping out at any visit equal to 1-(1-*d*)^1/(*T*-1)^ where *d* was the overall dropout rate, 0.25 or 0.40; and T-1 = 8 is the number of postbaseline visits when discontinuations were allowed to occur. Similarly, MAR and MNAR were also simulated using a Bernoulli distribution. A dropout model from the placebo-controlled period was built from the actual clinical trial data by applying logistic regression to repeated measures data with outcome R(t) = 1 if patients dropped at visit t and 0 otherwise. The covariates in the logistic regression model were baseline and change score from baseline. No dropouts were allowed at the first post-baseline visit. The logistic regression dropout model is log(pdrop(t)/(1-pdrop(t)) = a_0_+a_1_*x(t)+ a_2_* y(0), where y(0) is baseline severity score, x(t) is the change score from baseline, defined as: x(t) = y(t)-y(0), and the estimates of parameters a_0_ and a_1_ were based on the actual clinical trial data. For MAR, changes from baseline to the current visit affect probability of dropout at the *next* visit and a dropout indicator of one resulted in data from the next visit being dropped; for MNAR, a dropout indicator of one resulted in data from the current visit being dropped. Specifically, the parameter slope a_2_ was constrained to be at least 5 times as large as the baseline parameter a_1_, and all parameters were calibrated to ensure a desired overall dropout rate in the simulation. For MAR at 25% discontinuations, a_0_ = -3.5280, a_1_ = 0.06, and a_2_ = 0.004; for MAR at 40% discontinuations, a_0_ = -3.1176, a_1_ = 0.06, and a_2_ = 0.012. For MNAR at 25% discontinuations, a_0_ = -3.6, a_1_ = 0.04, and a_2_ = 0.004; for MNAR at 40% discontinuations, a_0_ = -3.3, a_1_ = 0.06, and a_2_ = 0.012. The dependence of discontinuations on observed outcomes inherent in MAR data (or on unobserved data for MNAR) is ensured by the non-zero coefficients a_2_.

The first MMRM analysis model used data from both the placebo-controlled period and the delayed-start period and included the terms baseline score, treatment, visit, and treatment-by-visit interaction. The second model used data from the delayed-start period only and included the same terms. Baseline score was included as a continuous variable; treatment and visit were categorical variables. The within-patient error was modeled using an unstructured covariance structure. Type III tests were used to test the effect of treatment at the final visit of the placebo-controlled period (month 18) and at the final visit of the delayed-start period (month 24).

The Type I error rates and power were tabulated for the five methods of noninferiority test procedures as described in Table 1. To assess the Type I error rate, the rejection rate under scenario 2 (assuming null hypothesis Δ_2_ ≤ 0.5 Δ_1_ is true) was calculated among the 2000 simulations. The nominal level was α = 0.10. To assess the power, the rejection rate under scenario 1 (assuming the alternative hypothesis Δ_2_ = Δ_1_ is true) was calculated among the 2000 simulations. The difference between estimated treatment least squares (LS) means and the simulated means from the complete data at months 18 and 24 were used to estimate the conditional biases.

## Results

### Noninferiority test procedures

#### Type I error rate.

The nominal Type I error rates are given in Table 3 for the five methods of noninferiority test procedures (Table 1). The estimated rejection rates with method 4 were within the simulation margin from the nominal Type I error rate of 0.1 (with 2000 runs, the simulation margin was calculated as 1.96*sqrt(0.1*(1–0.1)/2000), which was equal to 0.013) (Table 3). Method 2 was less conservative than method 4, but method 2 also never exceeded the nominal Type I error rate by more than 0.013. Methods 3 and 5 maintained Type I error rate; however, they appeared to be too conservative as they are much lower than the nominal level of 10%. Method 1 proved to be not valid since its Type I error rate substantially exceeded the nominal level of 10%.

**Table 3:** Type I error rates for candidate test procedures and various conditions of missingness mechanism, discontinuation rate, and data inclusion

	Placebo-Controlled plus Delayed-Start Period Data						Delayed-Start Period Data Only					
	MCAR		MAR		MNAR		MCAR		MAR		MNAR	
Method	25%	40%	25%	40%	25%	40%	25%	40%	25%	40%	25%	40%
1	0.399	0.370	0.384	0.360	0.391	0.354	0.395	0.396	0.383	0.356	0.383	0.341
2	0.101	0.101	0.102	0.096	0.100	0.098	0.100	0.118	0.100	0.093	0.101	0.089
3	0.043	0.049	0.043	0.042	0.043	0.046	0.045	0.050	0.040	0.035	0.042	0.041
4	0.087	0.087	0.087	0.086	0.084	0.084	0.083	0.094	0.084	0.077	0.087	0.076
5	0.027	0.033	0.028	0.025	0.032	0.028	0.031	0.035	0.026	0.020	0.028	0.023

MAR: missing at random; MCAR: missing completely at random; MNAR: missing not at random.

#### Power.

Table 4 lists the power of the five candidate noninferiority test procedures under the alternative hypothesis. Method 1 had the greatest power, followed by methods 2, 4, 3 and 5. While method 4 was associated with smaller Type I error rates than method 2, it had relatively less power than method 2.

**Table 4:** Power estimates for candidate test procedures under various conditions of missingness mechanism, discontinuation rate, and data inclusion

	Placebo-Controlled plus Delayed-Start Period Data						Delayed-Start Period Data Only					
	MCAR		MAR		MNAR		MCAR		MAR		MNAR	
Method	25%	40%	25%	40%	25%	40%	25%	40%	25%	40%	25%	40%
1	0.854	0.807	0.858	0.785	0.853	0.793	0.848	0.815	0.854	0.781	0.847	0.788
2	0.526	0.488	0.537	0.470	0.532	0.478	0.527	0.491	0.532	0.455	0.528	0.451
3	0.351	0.320	0.373	0.309	0.354	0.316	0.348	0.314	0.361	0.291	0.361	0.295
4	0.492	0.452	0.502	0.438	0.494	0.444	0.485	0.443	0.499	0.419	0.488	0.421
5	0.298	0.258	0.298	0.237	0.286	0.245	0.290	0.253	0.301	0.225	0.289	0.235

MAR: missing at random; MCAR: missing completely at random; MNAR: missing not at random.

### Bias in estimated least squares means

**Fig 2:**
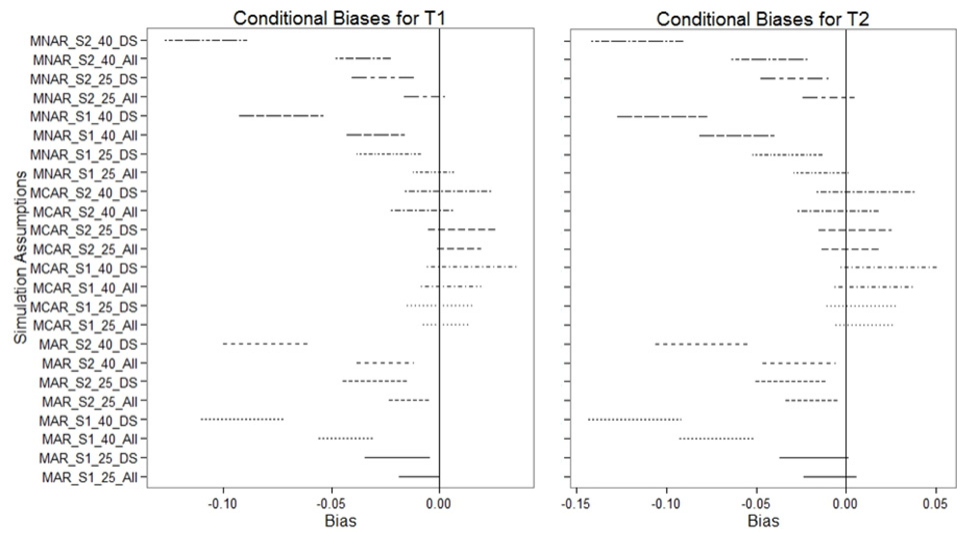
95% confidence intervals for conditional bias. 25: 25% discontinuation rate; 40: 40% discontinuation rate; All: all data from all randomized patients in both study periods; DS: delayed-start period data only; MAR: missing at random; MCAR: missing completely at random; MNAR: missing not at random; S1: scenario 1, disease-modifying treatment effect scenario; S2: scenario 2, symptomatic treatment effect scenario; T1: time 1, end of placebo-controlled period; T2: time 2, end of delayed-start period. Each line segment represents the 95% confidence interval for the conditional bias estimated across various missingness mechanisms, conditions of underlying drug effect, discontinuation rates, and modeling approaches.

Fig. 2 contains forest plots of the differences in conditional biases of estimated LS means between data analysis approaches (modeling over both placebo-controlled period and delayed-start period including all randomized patients [“All”] versus modeling over delayed-start period only [“DS”]) across various missingness mechanisms, conditions of underlying drug effect, and discontinuation rates. Bias results for MCAR data indicated no bias and were comparable for all sets of assumptions. However, bias results for both MAR and MNAR data showed larger biases when data were only modeled over the delayed-start period. These biases were greatest at higher rate of missingness of 40%. Additionally, smaller variances in estimated LS means of the “All” approach were observed compared with the variances in estimated LS means of the “DS” approach.

## Discussion

In this paper, we propose a novel method for conducting analyses in a delayed-start design to determine disease-modifying effects in AD. We assessed different approaches to test the hypothesis to determine whether the experimental treatment maintains a prespecified proportion of the treatment effect seen at the beginning of the delayed-start period (also known as the end of the placebo-controlled period) at the end of the delayed-start period. All five of these approaches to test this hypothesis use a noninferiority framework. The differences among the approaches arise from how the hypothesis is parameterized and the subsequent test statistics are derived.

The Type I error rates and power based on the simulation work were evaluated to determine the optimal method for the noninferiority test. Method 1 was clearly unacceptable because it failed to control the Type I error rates at the prespecified level, and methods 3 and 5 were unacceptable for lack of power. Method 4 had slightly lower Type I error and (accordingly) lower power compared with method 2. Because we consider it is more important to control the false positive rate, we believe method 4 is a more appropriate test procedure compared with method 2. In addition, method 4 (Δ_2–_0.5 Δ_1_ > 0) compares a quantity involving random variables to a constant, while method 2 (Δ_2_-Δ_1_ >-0.5 Δ_1_) has random variables on both sides of the inequality, which makes method 4 preferable. Based on these considerations, we concluded that method 4 is the optimal choice of the candidate noninferiority test procedures.

The simulation results for power appeared to be largely insensitive to the missingness mechanism (that is MCAR, MAR, or MNAR) or the study periods included in the analyses. However, they did show poorer performance associated with 40% dropout rate compared with 25% dropout rate. This speaks to the importance of making every effort in the conduct of the clinical trials to reduce dropouts.

We propose to conduct the noninferiority test using all available data from all randomized patients across both study periods in a single MMRM model. This is consistent with the general principle of using all available data at all time points to analyze longitudinal data in the estimation of treatment effects at endpoint. The simulation results showed that using all data from both the placebo-controlled and delayed-start periods (“ALL”) had smaller variances than using the delayed-start period only (“DS”) in estimating the outcomes. The differences in biases between the two approaches (“ALL” and “DS”) were minimal under MCAR scenarios regardless of dropout rates. However, the biases associated with “DS” were larger than those for “ALL” when the missingness mechanism was MAR or MNAR. In particular, the differences in biases between “ALL” and “DS” were the most pronounced when the dropout rate was high (40%).

As demonstrated by the results above, we propose that delayed-start analyses should be conducted by including all randomized patients and all available data over all time points in both the placebo-controlled and delayed-start periods. A single MMRM is fit to produce the estimate of the treatment effect at the beginning as well as at the end of the delayed-start period, Δ_1_ and Δ_2_, respectively. The noninferiority test can be carried out using method 4 (Table 1) as the test procedure.

**Fig 3:**
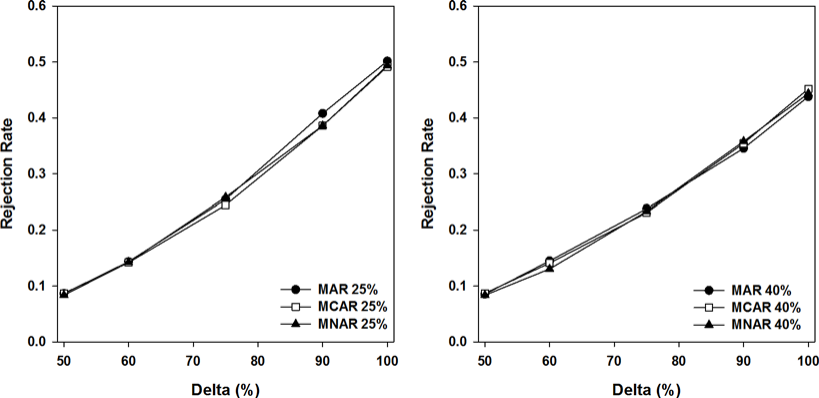
Power curves for underlying treatment scenarios for method 4 (Δ_2–_0.5 Δ_1_ > 0) for 25% patient dropout (left) and 40% patient dropout (right).

To further assess the characteristics of recommended method 4, we conducted additional simulation work by adding several underlying treatment scenarios, including Δ_2_ = 0.6 * Δ_1_, Δ_2_ = 0.75 * Δ_1_, and Δ_2_ = 0.9 * Δ_1_. The power curves based on these scenarios as well as the original scenarios of Δ_2_ = 0.5 * Δ_1_ and Δ_2_ = 1 * Δ_1_ are plotted in Fig. 3. The power curves represent simulated rejection rates for various scenarios of missingness (on the vertical axis) versus the proportion of treatment difference in the beginning of the delayed-start period (Δ_1_) that was retained at the end of the delayed-start period (Δ_2_) on the horizontal axis (delta = Δ_2_/ Δ_1_, %). As simulation results indicate, method 4 is tolerable to small departure from the null hypothesis of Δ_2_ = 0.5 * Δ_1_ regarding controlling the Type I error rate. As expected, it also shows that greater dropout rate is associated with lower power.

Rejection rate represents the proportion of trials in which the null hypothesis would be rejected among the 2000 simulations (that is, Type I error rate when Delta is equal to 50%). Delta represents the proportion of the treatment difference at the beginning of the delayed-start period (Δ_1_) that was retained at the end of the delayed-start period (Δ_2_) under various treatment scenarios tested in the simulations.

This proposed analysis methodology not only has desired statistical properties regarding Type I error rate and bias control as shown by the simulation results, it also circumvents the key methodological issues observed in ADAGIO. The ADAGIO analysis only included a proportion of the randomized patients, which led to a noted imbalance between the treatment groups included in the delayed-start analysis. This treatment imbalance significantly challenged the results and the interpretation of any perceived treatment effect. The proposal by Zhang et al. [10] has similar limitations as their nonnferiority analysis only includes data from the last 3 months of the delayed-start period.

Another concern with the approach used in the ADAGIO trial is that the method applied a noninferiority test comparing the two slopes between the delayed-start and early-start treatment groups using an absolute noninferiority margin. As a result, even though the two slopes converged at the end of the delayed start period, the test declared that noninferiority had been met. This clear contradiction was caused by the small magnitudes of both slopes. While the difference between the slopes was real and caused the two lines to cross each other, the magnitude of the difference was smaller than the fixed, absolute noninferiority margin. The problematic nature of using an absolute noninferiority margin has been reported in the literature [23]. The noninferiority test that we propose avoids the issue by using a relative margin.

The choice of noninferiority margin can be quite complex, especially in the field of AD where there have been very few examples, leading to a lack of specific regulatory guidance or consensus in the field. In general, a noninferiority margin should reflect clinical judgment about how much of the control effect should be preserved by ruling out the largest clinically acceptable loss [24]. For example, it has become usual practice for cardiovascular outcome studies to use 50% as the noninferiority margin to seek retention of 50% of the control effect. It is possible that a drug could have both symptomatic and disease-modifying effects. In this case, one would expect at least half of the overall treatment result is from the disease-modifying effect and this outcome should be preserved. Thus, we feel that a 50% noninferiority margin is reasonable and has clinical significance for a delayed-start trial in AD because a positive result using this margin would indicate that that no more than 50% of the treatment difference at the end of the placebo-controlled period would be lost at the end of the delayed-start period.

One additional advantage of the proposed methodology in this paper is that there is no need to assume linearity of disease progression. The previous proposals have called for comparison of the slopes between the delayed-start and early-start treatment groups as the mechanism to discern whether delayed-start patients have caught up with the early-start patients, such as in ADAGIO and Zhang et al.[10] The inherent assumption of slopes is that patients progress in a linear fashion. Much attention has been paid to the topic of linear progression (or a lack thereof) in AD [25,26]. Given an incomplete understanding in the field of the trajectory of AD progression, the consensus is that a linear progression cannot be assumed.

As it is not practical to cover every possible scenario and assumption, the simulation work conducted in this research was designed to include the most likely and reasonable scenarios and assumptions encountered in the setting of clinical trials under discussion.

FDA officials have acknowledged that results from an appropriately conducted delayed-start design could be interpreted and used to demonstrate a disease-modifying effect [27,28]. In addition, although the rasagiline advisory committee did not endorse a disease-modifying effect of rasagiline due to insufficient evidence based on ADAGIO, the committee members did reiterate that the method of delayed-start design is a valid option in demonstrating a disease-modifying effect [29]. Recently, FDA has issued a draft guidance on developing drugs for early stages of AD [30]. The draft guidance stated that a disease-modifying effect can be demonstrated through an effect on a biomarker in combination with clinical outcomes or it can be demonstrated by an alternative trial design, such as a delayed-start design, to show a lasting effect of early treatment on the disease course.

### Application to Phase 3 Randomized Clinical Trial Data

We applied the analytical methodologies we propose in this paper to data from patients with mild AD in Phase 3 studies for solanezumab, a humanized monoclonal antibody, for the treatment of AD. EXPEDITION and EXPEDITION2 (ClinicalTrials.gov numbers NCT00905372 and NCT00904683) were Phase 3, 18-month, placebo-controlled studies investigating the efficacy and safety of solanezumab in patients with mild to moderate AD. The trial protocols were approved by the ethical review board at each of the 206 study centers in 16 countries. These studies were conducted in accordance with the ethical principles that have their origin in the Declaration of Helsinki and that are consistent with good clinical practices (GCPs) and the applicable laws and regulations. A properly executed, signed Informed Consent Form (ICD) was obtained from each subject.The prespecified coprimary endpoints for EXPEDITION and EXPEDITION2 were the ADAS-Cog and the Alzheimer’s Disease Consortium Study Activities of Daily Living inventory (ADCS-ADL) at 18 months. The primary analysis results from these studies have been published by Doody and colleagues [31]. The primary objectives for EXPEDITION and EXPEDITION2 were not met, but a prespecified secondary analysis of pooled data from patients with mild AD in the studies did demonstrate a treatment signal [32]. EXPEDITION-EXT (NCT01127633) is an on-going, open-label extension study in patients who completed EXPEDITION or EXPEDITION2 (“feeder studies”). In EXPEDITION-EXT, patients who received solanezumab in the feeder studies remain on the active treatment (early start), while patients who received placebo in the feeder studies are switched to solanezumab (delayed start). During the delayed-start period, all patients receive solanezumab (thus named “open-label”); however both patients and site personnel remain blinded to patients’ original treatment assignment to solanezumab or placebo in the feeder studies. The primary objective in EXPEDITION-EXT is long-term safety. We tested the hypothesis of disease-modifying effect of solanezumab with the noninferiority test proposed in this paper, using pooled data from patients with mild AD in the EXPEDITION, EXPEDITION2, and EXPEDITION-EXT studies as the primary efficacy analysis for EXPEDITION-EXT. (Data analysis script available in S2 Table.) The delayed-start analysis methods described in this paper were communicated to FDA and were prespecified in the statistical analysis plan before database locks.

**Fig 4:**
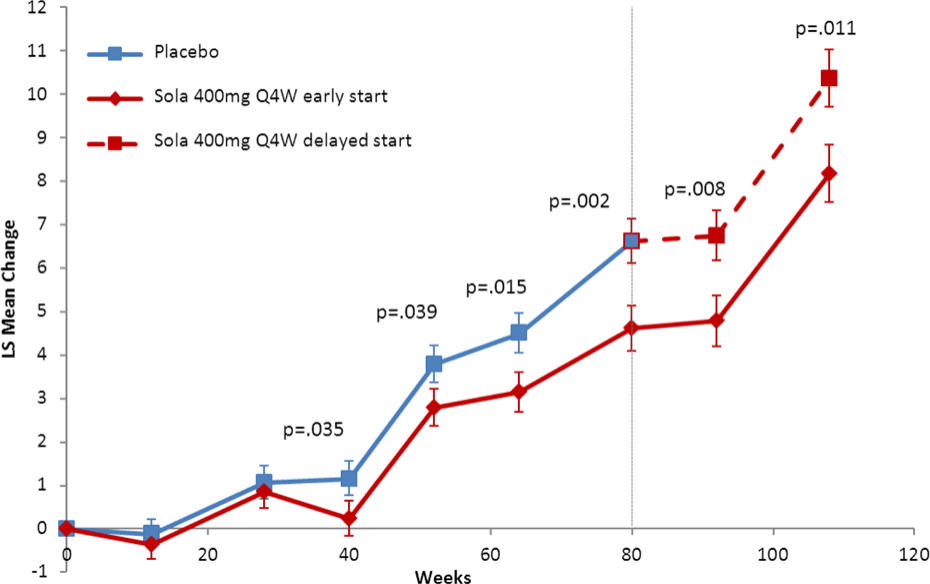
Delayed start analysis of ADAS-Cog14 scores in patients mild AD from the EXPEDITION, EXPEDITION2, and EXPEDITION-EXT studies of solanezumab. Abbreviations: ADAS-Cog_14_ = 14-item Alzheimer’s Disease Assessment Scale—Cognitive subscale; Q4W = every 4 weeks. Note: All p-values shown for values <.05 and endpoint. Error bars represent standard error. Dashed line indicates end of EXPEDITION and EXPEDITION-2 (feeder studies) and beginning of EXPEDITION-EXT.

In EXPEDITION and EXPEDITION2 combined, 660 patients with mild AD were randomized to placebo and 654 to solanezumab. Of these patients, 520 placebo-treated patients and 498 solanezumab-treated patients completed 18 months in the feeder studies. Based on the interim data cut off date for EXPEDITION-EXT on 20 June 2012, 240 placebo and 232 solanezumab patients had completed 28 weeks of treatment in the delayed-start period. Using one MMRM model across all of the data from feeder study baseline up to 28 weeks of the delayed-start period as proposed in this paper, the treatment difference between solanezumab and placebo for the ADAS-Cog_14_ at 28 weeks in EXPEDITION-EXT (Δ_2_) was 2.2 (p = .011); this was similar to the difference at the beginning of the delayed-start period (Δ_1_) (2.01, p = .002; the same time point as the end of the placebo-controlled studies; Fig. 4). The lower limit of the 90% confidence interval for the test statistic (Δ_2_ − 50% × Δ_1_) was 0.3697 which indicated that the noninferiority criterion was met, and the treatment difference in cognition between solanezumab and placebo observed at the end of the placebo-controlled studies was preserved at the end of the delayed-start period within a pre-defined margin (noninferiority criteria were also met using the other candidate test procedures in Table 1; see S1 Table). Thus, the results from the EXPEDITION studies suggest patients who received solanezumab rather than placebo during the double-blind studies had a benefit that could not be recovered by patients who began solanezumab later in EXPEDITION-EXT. This finding is thought to be consistent with a treatment effect that changes the underlying progression of AD.

In a delayed-start design, the placebo-controlled period should be sufficiently long to allow a disease-modifying drug to show an effect and the delayed-start period should be long enough to observe a symptomatic effect. Most clinical studies testing potential disease-modifying treatments have used 18 months for the placebo-controlled period, as in the EXPEDITION studies. Given that the half-life of solanezumab is approximately 28 days [33], a duration of 28 weeks (approximately 6 months) was chosen for the delayed-start period for the primary efficacy analyses of EXPEDITION-EXT to ensure that the duration of the period was over 5 half-lives and was thus adequate for delayed-start patients to achieve pharmacokinetic equilibrium. The literature also suggests that most symptomatic drugs reach peak effect in less than 6 months [34–36]. EXPEDITION-EXT is still ongoing and future analyses will demonstrate whether the effect persists beyond 28 weeks.

In summary, we believe this proposed method of delayed-start analysis is an improved methodology to better ascertain possible disease-modifying effects in AD, which can be interpreted to support the clinical meaningfulness of an AD treatment.

## Supporting Information

S1 Table Candidate test procedures for noninferiority test with results for EXPEDITION analyses. Note: For each procedure, the lower bound of the 1-sided 90% confidence interval must be greater than the specified threshold.
(DOCX)

S2 Table Data analysis script: delayed-start analysis. (DOCX)
