# Correction: A Novel Approach to Delayed-Start Analyses for Demonstrating Disease-Modifying Effects in Alzheimer’s Disease

**DOI:** 10.1371/journal.pone.0131338

**Published:** 2015-06-22

**Authors:** The PLOS ONE Staff

There is an error in the *H*_*a*_ equation in the second paragraph of the Methods section. The inequality symbol within this equation should read >. Please view the correct, complete equation here:

$$H_{a}:{∆}_{2}-{∆}_{1}>-0.5{∆}_{1}$$

The publisher apologizes for this error.
