# Supplementary material for: A Novel Approach to Delayed-Start Analyses for Demonstrating Disease-Modifying Effects in Alzheimer’s Disease
Source: PLoS One. 2015 Mar 17;10(3):e0119632. doi: 10.1371/journal.pone.0119632 (PMC4363486; doi:10.1371/journal.pone.0119632)
Supplement: S1 Table — Note: For each procedure, the lower bound of the 1-sided 90% confidence interval must be greater than the specified threshold. (DOCX) [file pone.0119632.s001.docx]

**Table S1.** Candidate test procedures for noninferiority test with results for EXPEDITION analyses

|  | **Lower bound of the 1-sided 90% CI:** |  | **Threshold** |
| --- | --- | --- | --- |
| (1) | $\Delta_{2}-\Delta_{1}= -0.501$ | > | -0.5x the upper bound of 1-sided 90% CI for $\Delta_{1}= -1.421$ |
| (2) | $\Delta_{2}-\Delta_{1}= -0.501$ | > | -0.5x the estimate for $\Delta_{1}= -1.005$ |
| (3) | $\Delta_{2}$ = 1.090 | > | 0.5x the estimate for $\Delta_{1}=1.005$ |
| (4) | $\Delta_{2}-{0.5\Delta}_{1}$ = 0.370 | > | 0 |
| (5) | $\Delta_{2}-\Delta_{1}= -0.501$ | > | -0.5x the lower bound of 1-sided 90% CI for $\Delta_{1}= -0.588$ |

Note: For each procedure, the lower bound of the 1-sided 90% confidence interval must be greater than the specified threshold.
