# Supplementary material for: A Novel Approach to Delayed-Start Analyses for Demonstrating Disease-Modifying Effects in Alzheimer’s Disease
Source: PLoS One. 2015 Mar 17;10(3):e0119632. doi: 10.1371/journal.pone.0119632 (PMC4363486; doi:10.1371/journal.pone.0119632)
Supplement: S2 Table — (DOCX) [file pone.0119632.s002.docx]

S2 Table. Data Analysis Script: Delayed-Start Analysis

options sasautos = ("&lumpath" "&bumpath" sasautos) formchar='|----|+|---+=|-/\<>*' NODATE NOCENTER NONUMBER NOBYLINE MPRINT PS=46 LS=133;

libname lzao "&inlib";

libname lzan "&adasin";

libname lzam "&lzam";

proc format ;

value vis

2 = 'Baseline'

6 = 'Week 12'

10 = 'Week 28'

13 = 'Week 40'

16 = 'Week 52'

19 = 'Week 64'

23 = 'Week 80'

24 = 'Week 92'

25 = 'Week 108';

run;

*** obtain data;

%macro sub(lib, adasitem);

data sub_&lib.;

set &lib..subjinfo;

%if &lib ne lzao %then %do;

if trtsort = 1 then therapy = 'LY400mg Q4W' ;

if trtsort = 2 then therapy = 'Placebo' ;

if trtindcblflg = 1 then concomuse = 'Yes' ;

else if trtindcblflg = 0 then concomuse = 'No' ;

else concomuse = '' ;

if subjitttr=1 and mmsetssevv1 = "Mild" ;

keep sdyid INVID SUBJID pinvid mmsetssevv1 ageyr TRT TRTSORT trtindcblflg therapy concomuse subjitttr ;

%end;

%else %do;

keep subjid;

%end;

format _all_;

run;

data adas_&lib.;

%if &lib ne lzao %then %do;

set &lib..adas;

where adasqsnum = "&adasitem" and 2 <= visid <= 23 and adasrn ne . and visidanl ne .;

keep sdyid invid subjid visid vistp adasblvaltr adaschgbltr adasblflgtr visidanl adasrn;

%end;

%else %do;

set lzao.adas;

if visid=4 then visidanl=24;

else if visid=8 then visidanl=25;

where adasqsnum = "&adasitem" and 1 < visid <= 8 and adasrn ne .;

keep subjid visid vistp visidanl adasrn;

%end;

format _all_;

run;

%mend;

%sub(lzam, ADASTS14);

%sub(lzan, ADASTS14);

%sub(lzao, ADASTS14);

data sub_feed;

set sub_lzam sub_lzan;

run;

proc sort data=sub_feed;

by subjid;

run;

proc sort data=sub_lzao;

by subjid;

run;

data pat;

merge sub_feed(in=f) sub_lzao(in=l);

by subjid;

if f;

run;

data adasfeed_base; set adas_lzam adas_lzan;

where adasblflgtr=1;

keep sdyid subjid adasblvaltr;

run;

proc sort data=adasfeed_base; by subjid;

run;

proc sort data=adas_lzao; by subjid;

run;

data adas_lzao; merge adasfeed_base(in=a) adas_lzao(in=b); by subjid;

if a and b;

ADASCHGBLTR = ROUND((ADASRN - ADASBLVALTR),.00000001);

run;

data adas;

set adas_lzam adas_lzan

adas_lzao;

if visidanl ne .;

run;

proc sort data=adas;

by subjid;

run;

data analysis;

merge adas(in=a) pat(in=b);

by subjid;

if b;

run;

*** limit multiple obs within visit;

proc sort data=analysis;

by invid subjid visidanl descending vistp;

run;

data analysis;

set analysis;

by invid subjid visidanl descending vistp ;

if last.visidanl;

drop visid;

run ;

proc sort data=analysis; by subjid visidanl;

run;

/* ADAS-Cog14 Score */

/****** raw mean at baseline ****/

proc means data = analysis n mean std ;

var adasrn ;

class visidanl therapy;

where adasblflgtr = 1 ;

ods output summary = adas_rwm_bs_sum;

run ;

/*************************** Raw mean by visit *****************************************/

ods output summary = adas_rwm_vis_sum ;

proc means data = analysis n mean std ;

var adasrn ;

class visidanl therapy;

where adasblvaltr ne . and pinvid ne . and visidanl ne . and mmsetssevv1 ne ''

and concomuse ne '' and ageyr ne . and therapy ne '' and adaschgbltr ne .;

run ;

data adas_rwm_vis_sum2 ;

set adas_rwm_bs_sum adas_rwm_vis_sum ;

nc = put(adasrn_n,5.0) ;

meanc = put(adasrn_mean,5.2) ;

stdc = put(adasrn_stddev,6.3) ;

drop nobs adasrn_n--adasrn_stddev ;

run ;

proc sort data = adas_rwm_vis_sum2 ; by visidanl therapy ; run ;

%macro rmmmrm;

ods listing close;

/********** Check Covariance structure for MMRM analysis: LsMean Change ***************************************/

%let typ = UN#TOEPH#ARH(1)#CSH#CS ;

%let z = 1 ;

%do %until ( &conver = Yes or %qscan(&typ,&z,'#') eq %bquote( ) ) ;

%let cov = %scan(&typ,&z,'#') ;

/* MMRM analysis: LsMean Chage by visit */

ods output ConvergenceStatus = Convergence ;

proc mixed data = analysis(where=(adaschgbltr ne .)); * scoring = 2;

class pinvid therapy visidanl subjid concomuse;

model adaschgbltr = adasblvaltr pinvid therapy visidanl therapy*visidanl concomuse ageyr / DDFM = KENWARDROGER ;

repeated visidanl / sub = subjid(pinvid) type = &cov ;

lsmeans therapy*visidanl /slice=visidanl pdiff cl ;

run ; quit ;

%if %sysfunc(exist(Convergence)) %then %do ;

DATA _null_;

set Convergence;

if status = 0 and pdh = 1 then call symput ('conver' , 'Yes') ;

else call symput ('conver', 'No') ;

RUN ;

%end ;

%else %do ;

data _null_;

%put 'Data Convergence not created or not good convergence' ;

%let conver = No ;

run ;

%end;

%if &conver = No %then %goto next ;

%next: %let z = %eval(&z+1) ;

%end ;

/* MMRM analysis: LsMean Chage by visit

* ( keep = visidanl therapy estimate stderr upper lower ) */

ods output LSMeans = adas_chgls

diffs = adas_chgdf ( where = ( visidanl = _visidanl ) )

Tests3=tests3

estimates=_est;

proc mixed data = analysis(where=(adaschgbltr ne .)); * scoring = 2;

class pinvid therapy visidanl subjid concomuse;

model adaschgbltr = adasblvaltr pinvid therapy visidanl therapy*visidanl concomuse ageyr / DDFM = KENWARDROGER ;

repeated visidanl / sub = subjid(pinvid) type = &cov r rcorr;

lsmeans therapy*visidanl /slice=visidanl pdiff cl cov ;

estimate 'LY effect at visit 23(Delta 1)' therapy -1 1 therapy*visidanl 0 0 0 0 0 -1 0 0 0 0 0 0 0 1 0 0 ;

estimate 'LY effect at visit 25(Delta 2)' therapy -1 1 therapy*visidanl 0 0 0 0 0 0 0 -1 0 0 0 0 0 0 0 1 ;

run ; quit ;

data est T1 T2;set _est;

LowerB90=estimate-1.282*stderr;*1-sided 90% lower bound1.645, 1.282;

upperB95=estimate+1.96*stderr;*2-sided 95% lower bound;

LowerB95=estimate-1.96*stderr;*2-sided 95% lower bound;

if label='LY effect at visit 23(Delta 1)' then do; indication="T1"; output T1 est;end;

if label='LY effect at visit 25(Delta 2)' then do; indication="T2"; output T2 est; end;

run;

data _T1; set T1;

P1=probt;

T1=estimate;

T1_Ul95=upperB95;

T1_Ll95=LowerB95;

T1_Ll90=lowerB90;

keep T1_Ul95 T1 P1 T1_Ll95 T1_Ll90;

run;

data _T2; set T2;

P2=probt;

T2=estimate;

T2_Ll90=lowerB90;

T2_Ll95=LowerB95;

keep T2_Ll90 T2 P2 T2_Ll95;

run;

data test31; set adas_chgls;

indication='';

if therapy = 'LY400mg Q4W' and visidanl=23 then indication='B';

if therapy = 'Placebo' and visidanl=23 then indication='C';

if therapy = 'LY400mg Q4W' and visidanl=25 then indication='D';

if therapy = 'Placebo' and visidanl=25 then indication='E';

if indication='' then delete;

drop cov1-cov5 cov7 cov9-cov13 cov15;

run;

data vb vd vc ve vbd vbc vbe vcd vce vde; set test31;

if indication='B' then do; varB=cov6; output vb ; end;

if indication='D' then do; varD=cov8; output vd ; end;

if indication='C' then do; varC=cov14; output vc ; end;

if indication='E' then do; varE=cov16; output ve ; end;

if indication='B' then do; covBD=cov8; output vbd ; end;

if indication='B' then do; covBC=cov14; output vbc ; end;

if indication='B' then do; covBE=cov16; output vbe ; end;

if indication='C' then do; covCD=cov8; output vcd ; end;

if indication='C' then do; covCE=cov16; output vce ; end;

if indication='D' then do; covDE=cov16; output vDE ; end; run;

DATA vb; SET vb;keep varB;RUN ;

DATA vD; SET vD;keep varD;RUN ;

DATA vC; SET vC;keep varC;RUN ;

DATA vE; SET vE;keep varE;RUN ;

DATA vbD; SET vbD;keep covBD;RUN ;

DATA vbC; SET vbC;keep covBC;RUN ;

DATA vbE; SET vbE;keep covBE;RUN ;

DATA vCD; SET vCD;keep covCD;RUN ;

DATA vCE; SET vCE;keep covCE;RUN ;

DATA vDE; SET vDE;keep covDE;RUN ;

DATA SE3; merge _T2 _T1 vb vd vc ve vbd vbc vbe vcd vce vde;

nim=0.5;

* concise version of se;

se=sqrt(varD+varE+(1-nim)**2*(varB+varC)-2*(1-nim)*(covbd+covce));

* full version of se;

se2=sqrt(varD+varE+(1-nim)**2*(varB+varC-2*covbc)-2*covde-2*(1-nim)*(covbd+covce-covcd-covbe));

L90_T2_1_nimT1=T2-(1-nim)*T1-1.282*se; * 1-sided 90% lower bound1.645;

*compute the CI for T2-50%T1;

L90_T2_1_nimT12=T2-(1-nim)*T1-1.282*se2;

format L90_T2_1_nimT12 L90_T2_1_nimT1 8.4 se2 8.3;

run;

data ci; set se3;

therapy="LY400mg Q4W";

visidanl=23;

keep therapy visidanl L90_T2_1_nimT12 se2;

label L90_T2_1_nimT12="Computed CI for T2-0.5T1";

run;

data adas_chgls2 ;

set adas_chgls ;

lsmeanchg = compress(put(ESTIMATE,8.2)) ;

stderrc = compress(put(stderr,8.3)) ;

drop estimate stderr ;

run ;

data adas_chgdf2 ;

set adas_chgdf ;

pval = compress(put(probt,6.3)) ;

ci = "("||COMPRESS(PUT(LOWER,8.2))||", "||COMPRESS(PUT(UPPER,8.2))||")" ;

lsmeandiff = compress(put(ESTIMATE,8.2)) ;

keep visidanl ci lsmeandiff pval ;

run ;

/******************** Combine the results ****************************************************/

proc sort data = adas_chgls2 ; ; by visidanl therapy ; run ;

proc sort data = adas_chgdf2 ; by visidanl ; run ;

data adas_prefinal ;

merge adas_rwm_vis_sum2 adas_chgls2 ;

by visidanl therapy ;

run ;

data adas_prefinal2 ;

merge adas_prefinal adas_chgdf2 ;

by visidanl ;

run ;

/* Consolidate the data for final dataset */

proc sort data = adas_prefinal2 ; by visidanl therapy ; run ;

data adas_prefinal3 ;

retain viswk therapy nc meanc stdc lsmeanchg stderrc lsmeandiff pval ci ;

set adas_prefinal2 ;

by visidanl ;

viswk = put(visidanl, vis.);

if therapy = "Placebo" then do ;

lsmeandiff = '' ;

pval = '' ;

ci = '' ;

end ;

run ;

proc sort data = adas_prefinal3 out = adas_final ; by visidanl descending therapy ; run ;

data adas_final; merge adas_final(in=a) ci(in=b); by visidanl descending therapy;

if a;

run;

/* Create report */

ods listing;

title1 "%justify1(ttln=1, text=Summary of ADAS-Cog14 - Repeated Measures Analysis)" ;

title2 "%justify1(ttln=2, text=Intent-to-Treat Population/MMSE Severity at Visit1 Mild)" ;

title3 "%justify1(ttln=3, text=%str(Study H8A-MC-LZAM, Study H8A-MC-LZAN and Study H8A-MC-LZAO))";

title4 ' ' ;

footnote1 "Abbreviations: CI = confidence interval; LS Mean = least squares mean; SD = standard deviation; SE = standard error." ;

footnote2 "*a - Mixed Model Repeated Measures (MMRM): " ;

footnote3 " Change = Baseline + Pooled Investigator + Treatment + Visit + Treatment*Visit" ;

footnote4 " + Baseline ACHEI/Memantine use + Baseline Age; Covariance Structure: &cov." ;

footnote5 "T2: the difference between Placebo and LY at week 108, T1: the difference between Placebo and LY at week 80" ;

footnote6 "If the lower bound of the 1-sided 90% CI for T2-0.5T1 > 0, then non-inferiority is met.";

footnote7 "Program: Home/lillyce/prd/ly2062430/h8a_mc_lzao/intrm1/programs_stat/adhoc_analysis/rmdelay1_adas14.sas" ;

footnote8 "Output: Home/lillyce/prd/ly2062430/h8a_mc_lzao/intrm1/programs_stat/tfl_output/adhoc_analysis/rmdelay1_adas14.rtf" ;

footnote9 "Data:Home/lillyce/prd/ly2062430/h8a_mc_lzan/final/data/shared/ads,Home/lillyce/prd/ly2062430/h8a_mc_lzam/final/data/shared/ads" ;

footnote10 "Data: Home/lillyce/prd/ly2062430/h8a_mc_lzao/intrm1/data/shared/ads" ;

proc report data = adas_final NOWD NOCENTER SPLIT = "~" HEADLINE HEADSKIP MISSING ;

columns ('--' viswk therapy nc

('Raw Score' '--' meanc stdc )

('Change from Baseline' '--' lsmeanchg stderrc lsmeandiff pval ci L90_T2_1_nimT12 se2) ) ;

define viswk / display 'Week' group left width = 8 order=data ;

define therapy / display 'Treatment' width = 12 ;

define nc / display 'N' width = 5 center ;

define meanc / display 'Mean' width = 5 left ;

define stdc / display 'SD' width = 6 center ;

define lsmeanchg / display 'LS Mean~Change' center width = 8 ;

define stderrc / display 'SE' width = 6 center ;

define lsmeandiff / display 'LS Mean~Difference' center width = 10 ;

define pval / display 'P-Value*a' center width = 9 ;

define ci / display '95% CI' width = 14 center ;

define L90_T2_1_nimT12/display "Lower Bound~of~CI(T2-0.5T1)" width = 12 center;

define se2/display "SE for CI~(T2-0.5T1)" width = 10 center;

break after viswk / skip ;

run ;

%mend;

%envt5(reportname=rmdelay1_adas14);
